# 

**Published:** 1983-10

**Authors:** 


OCTOBER 1983
VOLUME 98 (iv)
NUMBER 368
Published Quarterly
Annual Subscription £8 Post Free

### BRISTOL MEDICO-CHIRURGICAL JOURNAL

Established 1883

### BRISTOL MEDICO-CHIRURGICAL JOURNAL

Incorporating the Medical Journal of the South West

202. Correspondence

203. Programmes of Societies

205. Examination Results

Printed in Great Britain by John Wright & Sons (Printing) Ltd, at The Stonebridge Press, Bristol

### Editorial Committee

EDITOR
*Mr. M. G. Wilson*
*Litfield House, Clifton, Bristol BS8 3LS*

ASSISTANT EDITOR
*Dr. C. R. Tribe*
*Southmead Hospital, Bristol BS10 5NB*

MEMBERS
*Dr. M. B. Lennard*
*Dr. D. W. Wright*
*The Hon. Treasurer of the Society*
*The Hon. Secretary of the Society*

SECRETARY
*Mr. B. P. Jones, The Library, Medical School, University Walk, Bristol BS8 1TD*

### Correspondents

*Dr. J. Jancar*
*Dr. H. G. Mather*
*Professor G. M. Stirrat*
*Dr. J. L. G. Thomson*
*Dr. C. R. Tribe*
*Dr. M. J. Whitfield*
*Professor R. C. N. Williamson*
*Dr. D. W. Wright*

### Bristol Medico-Chirurgical Society

PRESIDENT 1983/84
*Dr. N. C. Tricks*

PRESIDENT ELECT

*Dr I. S. Bailey*

HONORARY SECRETARY
*Dr. H. M. Norman*
*Hillside, Vicarage Lane, Barrow Gurney*

HONORARY TREASURER
*Dr. J. B. Penry.*
*Southmead Hospital, Bristol BS10 5NB*

The Society was founded in 1874. In 1883 publication of the Journal began and has continued quarterly ever since then. During the years 1949 to 1962 it appeared under the title of 'The Medical Journal of the South West'.

### Notice to Contributors

*The Journal is especially concerned to provide a place for the recording of medical thought, interests and practice in and around Bristol. It therefore welcomes articles that, as well as being of immediate interest to members, will document the local and contemporary medical scene for our successors.*

*Original articles are preferred in the form proposed by the International Committee of Medical Editors (The Vancouver System) and published as an article in the British Medical Journal in 1979 (Br. Med. J.* **1**, *532–535). This has been reprinted as a pamphlet and may be obtained from the Editor of the B.M.J., B.M.A. House, Tavistock Square, London WC1H 9JR (enclose 50p).*

*Also welcome are letters to the Editor, Book Reviews, news of important appointments and events, etc.*

### Programmes of Societies, 1983–84

### BRISTOL MEDICO-CHIRURGICAL SOCIETY

Meetings
1983–1984

1983

Wednesday 12th October
A.G.M. Presidential Address
*Medical School*

Wednesday 9th November
Long Fox Memorial Lecture
*Medical School*

Wednesday 14th December
The Scrope Davies Papers
Mr. Martin Davies
*Southmead*

1984

Wednesday 11th January
'Problems of Sight in the Cradle and in Senescence'
Mr. Gordon Catford
*Jenner Centre, BRI*

Wednesday 8th February
A symposium on an ethical subject chaired by Dr. Sandy Macara
*Medical School*

Wednesday 14th March
To be arranged

Wednesday 18th April
Clinical meeting and supper at University of Bristol Veterinary College, Langford

Wednesday 9th May
Annual Summer Meeting at RNLI Museum, Bristol when Dr. Geoff Wood will speak on his work as a Lifeboat Medical Officer on the Lizard

### COSSHAM MEDICAL SOCIETY

Meetings
1983–1984

All meetings at Frenchy Postgraduate Centre, 8.30 p.m.

1983

Tuesday 4th October
Presidential Address
Dr. David Skelton

Tuesday 1st November
'Old-fashioned Obstetrics'
Mr. Peter Niven

Saturday 26th November
Annual Cocktail Party, 8 p.m. at 'High Croft', Bury Hill, Winterbourne Down

Tuesday 6th December
'The Three Peaks Yacht Race'
Dr. Rob Hawarth

1984

Tuesday 10th January
'An Illustrated Disconnected Ramble through old and not so old Bristol'
Dr. James Briggs

Tuesday 7th February
'Witchcraft and Herbal Medicine in Developing Countries'
Dr. Richard Evans

Tuesday 6th March
'The Wild Far West'
Dr. A. C. Hunt

Tuesday 3rd April
A.G.M.
'An Oral Surgeon's lot'
Mr. Gerry Pell

### BRITISH MEDICAL ASSOCIATION
### BRISTOL DIVISION

Meetings
1983–1984

1983

Wednesday 19th October. 8 p.m.
Annual Meeting:
Induction of President
for 1983–1984: Dr. Michael Simmonds
*Southmead Postgraduate Centre*

Sunday 16th October. 11 a.m.
St. Luke's Service
Preacher: Rev. Paul Berg
*Christchurch, Clifton*

Friday 11th November. 7.30 for 8 p.m.
Annual Dinner
Principal Guest and Speaker
Mr. Charles Clarke
Chairman Bristol and Weston H.A.
*The Red Lodge*

Wednesday 7th December. 8.30 p.m.
'The Relationship of Dentistry and Medicine'
Professor Crispin Scully
University of Bristol
*School of Nursing, Bristol Royal Infirmary*

1984

Friday 3rd February
(Subject to be announced)
Dr. Dennis Pereira Gray
S. W. Regional Postgraduate Adviser in General Practice
*Southmead Postgraduate Centre*

Wednesday 21st March
(Subject to be announced)
Dr. A. W. Macara
Chairman of Central Ethical Committee B.M.A. and Member of General Medical Council
*Southmead Postgraduate Centre*

Friday 15th June
Summer Reception
*The Pump Room, Bath*

### BRISTOL MEDICO LEGAL SOCIETY

Meetings
1983–1984

The meetings will be held in the School of Nursing at the Bristol Royal Infirmary and a buffet supper will be available from 6.30 p.m.

1983

Thursday 20th October. 8 p.m.
Don't Die Abroad – the problems of investigating death from overseas

Dr. Michael Green
*Department of Forensic Medicine, University of Leeds*

Thursday 17th November. 8 p.m.
Dialect and Voice Identificiation
Mr. Stanley Ellis, M.A., M.I.L.
*The School of English, University of Leeds*

1984

Thursday 19th January. 8 p.m.
The Shroud of Turin
Professor James Cameron, F.R.C.Path.
*Department of Forensic Medicine, University of London*

Friday 24th February
Banqueting Room, Council House, Bristol
Annual Dinner
Mr. Gilbert Gray, Q.C.

Thursday 15 March. 8 p.m.
Drug Abuse
Dr. Martin Gay, F.R.C.Psych.
Consultant Child Psychiatrist

Thursday 17th May. 8 p.m.
Members' Papers

Friday 8th July
Summer Social Gathering

### University of Bristol Faculty of Medicine

### THE DEGREE OF DOCTOR OF MEDICINE
**June 1983**

Anna Frances GLASIER
Erle Rodney LITTLEWOOD
Andrew Brent TULLO

### THE DEGREE OF DOCTOR OF PHILOSOPHY
**June 1983**

Paul Nicholas BROWN
Claire Alexandra SAVILLE

### FINAL EXAMINATION FOR THE DEGREES OF M.B., Ch.B.
**June 1983**

PASS

*With Honours*

Roger William Grenville BEER – with Distinction in Clinical Pathology
Anthony John Barrett EMMERSON – with Distinction in Surgery
Katherine PENDRY – with Distinction in Mental Health
Peter William RICHARDS – with Distinctions in Medicine, in Child Health and in Obstetrics and Gynaecology
Clare Margaret ROBERTSON – with Distinction in Child Health
Fiona Mary SHORT – with Distinction in Community Health
Steven Alan WEBBER – with Distinctions in Community Health and in Mental Health

*Pass*

Elizabeth Marguerite AGNEW – with Distinction in Mental Health
Margaret Clare AINGER
Susan Jennifer ARMSTRONG
Harpreet ARSHI
Ian Richard BAILEY
Stella Jane BAILEY
Timothy Graham BARRON
John Stephen BARTON
Jacqueline Anne BENNETT
Heather Angela BLACK
Yvonne Helen BOHM
David Carson BOYD
Jennifer Laura BROCKIS
Philip John Villiers BRYSON
Heather Mary BURKE
Margaret BUSHBY
Nicholas David BUXTON
Simon Charles CAYRE
Peter Douglas COLLINS
Tessa COOK – with Distinction in Mental Health
Alison Louise COOPER
Peter John CORMIE
Nicholas Robert Laurence COWAN
Jennifer Frances CROSS
Isaac DAUREEAWO
Jennifer Anne DAVIES
Judith Anne DAVIES
Philip Mark DYER
Jennifer Patricia EADIE
Hiliary Ann EDDOWES
Steven John ELLIS
Carolyn Rosalie FANG
Marwan Omar FAROUK
Anne Lesley FORBES
Christine Anne FOSTER
Shirley Ann GRACIAS
Helen Claire GRIFFITHS
Deborah Jane HANCOX
Jane Elizabeth HARCOURT
Sian Mererid HARRIS
Susan Joan HARRISON
Virginia Elizabeth HEPBURN
Claire Suzanne HEWETT
Matthew Anthony Richard HOGHTON
David Thomson HOWE
Gillian Mary JARVIS – with Distinction in Community Health
Paul Terrence JOHNS
Matthew Anselm JOHNSON
Sukhvinder Singh KASBIA
Philip James George KIRBY
David Christopher KIRRAGE
Mark Alexander KORDAN
Theodore KYRIAKIDES
Sarah Jane LACK
Azim Abdulkarim LAKHA
Robert John LAMBOURN
David Jeremy LANSLEY
John Allan LETHEM
Melanie Anne MACKINTOSH
Timothy Raymond MAGEE

Robert David MAYER – with
Distinction in Mental Health
Clare Alison MINTY
Robert Anthony MORGAN
Caroline Elizabeth MORLEY
Gail Margaret MORTON
Margaret Caroline NAYSMITH
Besim Evren NERELI
Hilary Ann NEVE
Sheelin Jane NEWTON
Teresa Felicity NICHOLSON
Richard Simon NOBLE
Jeremy Paul NOLAN
Vernon John PARFITT
Zoe Anne PINTO
Christopher Mark POLGE
Jill POSTINS
Sandra Carole RACHMAN
Angela Carolyn RAMSAY
Simon Guy RAY
Christopher James REE
Midhat Nafis SIDDIQI
David James SMALL
Janet Marie SMITH
Mohammed Shikh SOBEH
William Eric SPONSEL
Alison Mary SQUIRE
Jacqueline Margaret STOKES
Michael Francis Scott TAYLOR
Margaret Anne THICKETT
Kathleen Anne THOMAS
Elinor Mary THOMPSON
Emma Carol Margaret THOMPSON
David Ian TOVEY
Jonathan James Andrew TRAIN
Harriet Teresa Wynn TULLBERG
Colin TURNER
Jessica Cornelia St. Helier TWENEY
Carl Stuart VENN
Nicholas John VINER
Sarah Joanna WATERS
Anthony Charles WILCOCK
Mark Anthony WILCOX
Gillian Susan Lizbeth WILSON
Madeleine Ann WILSON
Kenneth Albert WOOD
Peter Trevor WOOD
Richard Neil Ashley WOOD
Stephanie Joan WOOD
Melanie Joy WOOLGAR
Sophia Ruth WRIGLEY
Ian Henry YELLOWLEES

